# Identification and Expression of miRNAs Related to Female Flower Induction in Walnut (*Juglans regia* L.)

**DOI:** 10.3390/molecules23051202

**Published:** 2018-05-17

**Authors:** Li Zhou, Shaowen Quan, Hang Xu, Li Ma, Jianxin Niu

**Affiliations:** 1 Department of Horticulture, College of Agriculture, Shihezi University, Shihezi 832003, Xinjiang, China; yuebanwan7@126.com (L.Z.); qsw@stu.shzu.edu.cn (S.Q.); xuhang1968@163.com (H.X.); maximaxi7@126.com (L.M.); 2 Xinjiang Production and Construction Corps Key Laboratory of Special Fruits and Vegetables Cultivation Physiology and Germplasm Resources Utilization, Shihezi 832003, Xinjiang, China

**Keywords:** female flower induction, microRNA, walnut (*Juglans regia* L.), high-throughput sequencing, RT-qPCR

## Abstract

Flower induction is an essential stage in walnut (*Juglans regia* L.) trees, directly affecting yield, yield stability, fruit quality and commodity value. The objective of this study was to identify miRNAs related to female flower induction via high-throughput sequencing and bioinformatics analysis. A total of 123 miRNAs were identified including 51 known miRNAs and 72 novel miRNAs. Differential expression was observed in 19 of the known miRNAs and 34 of the novel miRNAs. Twelve miRNAs were confirmed by RT-qPCR. A total of 1339 target genes were predicted for the differentially expressed miRNAs. The functions of 616 of those target genes had been previously annotated. The target genes of the differentially expressed miRNAs included: (i) floral homeotic protein *APETALA 2 (AP2)* and ethylene-responsive transcription factor *RAP2-7* which were targeted by jre-miRn69; (ii) squamosa promoter-binding protein 1 (*SPB1*) and various *SPLs* (squamosa promoter-binding-like protein) which were targeted by jre-miR157a-5p; (iii) various hormone response factors which were targeted by jre-miR160a-5p (*ARF18*) and jre-miR167a-5p (*ARF8*) and (iv) transcription factor *SCL6* which was targeted by jre-miR171b-3p, jre-miRn46 and jre-miRn49. The KEGG pathway analysis of the target genes indicated that the differentially expressed miRNAs were mainly enriched to ubiquitin mediated proteolysis, RNA degradation and various carbohydrate metabolism pathways. Many miRNAs were detected in *J. regia* during female flower induction. Some miRNAs (jre-miR157a-5p, jre-miR160a-5p, jre-miR167a-5p, miR171b-3p jre-miRn69 and jre-miRn49) were involved in female flower induction. The results of this experiment will contribute valuable information for further research about the function of miRNAs in flower induction of *J. regia* and other fruit trees.

## 1. Introduction

Walnut (*Juglans regia* L.) is an economically important nut tree, with more than 7000 years of evolutionary and domestication history in China [1,2]. The development of *J. regia* flowers begins with flower induction, flower initiation, and flower differentiation during the first growing season and ends with blooming in the second growing season. Flower induction involves the anatomical and morphological transition from vegetative meristems to reproductive meristems [3]. The date, intensity and quality of flower induction and differentiation directly influence the fruit tree’s early fruiting, fruiting stability, fruit quality and commodity value. Flower induction in woody trees is closely related to the time when new shoots stop growing [4]. In *J. regia*, the induction of female flowers occurs 3 to 7 weeks after medium and short branches cease vegetative growth. Initiation and differentiation of female flowers occur 4 to 10 weeks after the branches stop growing [5]. Flower induction and differentiation is a highly complex physiological, biochemical and morphogenetic process, which is influenced by various factors such as carbohydrate, starch, endogenous hormones, polyamines, metabolism of nucleic acids. In *Arabidopsis*, flowering involves five major pathways, including the vernalization pathway, the gibberellic acid (GA) pathway, the photoperiod pathway, the autonomous pathway and the aging pathway regulated by miRNAs [6].

MicroRNAs (miRNAs) are small RNAs (20–24 nucleotides) that play important roles in plant growth, flower development and the response to biotic and abiotic stresses. The first miRNAs to be discovered, lin-4 and let-7, regulate juvenile-to-adult transition in *Caenorhabditis elegans* [7,8]. In plants, miRNAs play essential roles in various pathways controlling flower development, serving either to inhibit or to promote reproductive phase transition. The expression of the MIR156 and MIR172 families controls the transition from the juvenile to the adult phase and from the vegetative to the reproductive phase [9]. Over-expression of miR156 prolongs the expression of juvenile vegetative traits and delays flowering in both *Arabidopsis* and *Zea mays*. In contrast, over-expression of miR172 accelerates flowering in *Arabidopsis* [10,11,12,13]. The MIR159, MIR319, MIR390 and MIR399 families have also been shown to influence flowering time. The MIR159 family has been implicated to play a role in the GA pathway, which promotes flowering under non-inductive conditions in *Arabidopsis* [14]. Over-expression of miR319 results in a late-flowering phenotype under long-day conditions in *Arabidopsis* [14,15]. MiR390 delays flowering by inhibiting the expression of its target transcription factor *ARF3/4* [16]. The miR399–*PHO2–IPS1* module was reported to regulate flowering time in response to ambient temperatures [17]. In precocious trifoliate orange, miR156/157and miR159 both showed significant down-expression from the juvenile to the adult stages compared with wild type trifoliate orange [18]. The same study showed that miR156 and miR172 had opposite expression patterns: miR156 expression declined from the juvenile to the adult stage whereas miR172 expression increased. In *Carya cathayensis*, the expressions of cca-miR156, cca-miR160, cca-miR167, cca-miR172 and cca-miR394 were differentially up-regulated during flower development, indicating that these miRNAs play important roles in flower induction [19].

However, the miRNAs involved in female flower induction and differentiation in *J. regia* remain unknown. In this study, we deep sequenced the small RNAs in walnut cultivar ‘Xinxin 2’ (*J. regia* cv. Xinxin 2). The short sequences were used to predict conserved and novel miRNAs. To identify the potential target genes of the miRNAs, the mRNA transcriptome was also created. The objective of this study was to discover miRNAs and their expression patterns during female flower induction in *J. regia*.

## 2. Results

### 2.1. Analysis of miRNA Sequences

**Figure 1:**
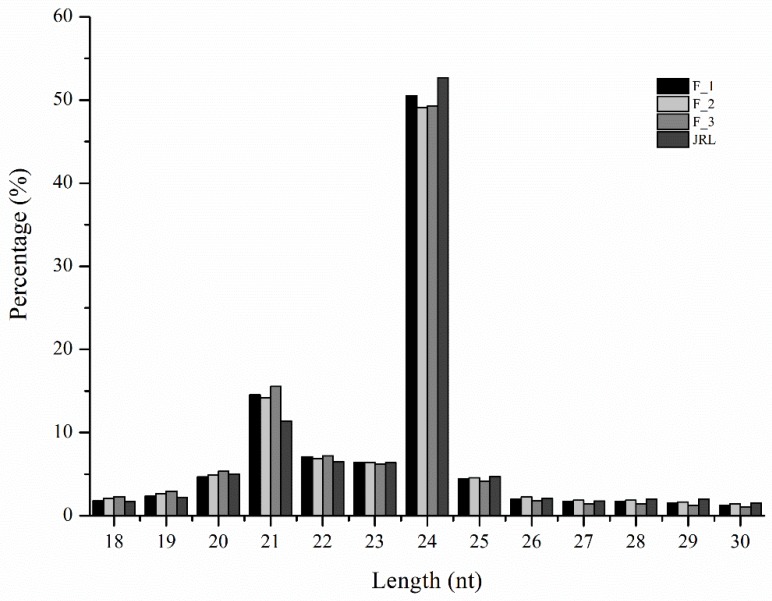
Length distribution and frequency of sRNAs in the four *J. regia* libraries. F_1, female flower buds before flower induction; F_2, female flower buds during flower induction; F_3, female flower buds after flower induction; JRL, leaf buds during flower induction.

We constructed three small RNA libraries using female flower buds collected at different stages of flower development: (i) before flower induction (F_1); (ii) during flower induction (F_2); and (iii) after flower induction (F_3). A fourth library was constructed using leaf buds collected during flower induction (JRL). A total of 47,462,626 raw reads were obtained from the four libraries (Table 1). After discarding the low quality reads and adapters, 46,579,260 clean reads were obtained. Clean reads with a length of 18 to 30-nt were selected for further analysis. A total of 39,321,617 small RNAs (sRNAs) were identified, with 10,765,695 sRNAs in F_1, 10,020,557 sRNAs in F_2, 10,094,699 sRNAs in F_3 and 8,440,666 sRNAs in JRL. The sRNAs were typically 21- to 24-nt in length (Figure 1). Among these sequences, 24-nt sRNAs were the most abundant sRNAs in all four libraries, accounting for 50.53% of the total reads in F_1, 49.11% in F_2, 49.25% in F_3, and 52.68% in JRL. The 21-nt sRNAs were second most abundant, accounting for 14.54% of the total reads in F_1, 14.19% in F_2, 15.56% in F_3, and 11.38% in JRL. Interestingly, there were more 24-nt sequences in the leaf buds (JRL) than in the female flower buds (F_1, F_2, and F_3). In contrast, there were fewer 21-nt sRNAs in the leaf buds (JRL) than in the female flower buds (F_1, F_2, and F_3). A total of 17,710,434 reads from the four libraries were mapped to the *J. regia* transcriptome. The mapped sRNAs were classified into several RNA categories, including known miRNA, novel miRNA, rRNA, tRNA, small nuclear RNAs (snRNA), small nucleolar RNAs (snoRNA), and ta-siRNA (Table 1). An average of 837,129 sRNAs (18.79% of the mapped sRNAs) were identified as known miRNAs, and an average of 92,751 reads (2.06%) were identified as novel miRNAs.

### 2.2. Identification of Known miRNAs in J. regia

**Figure 2:**
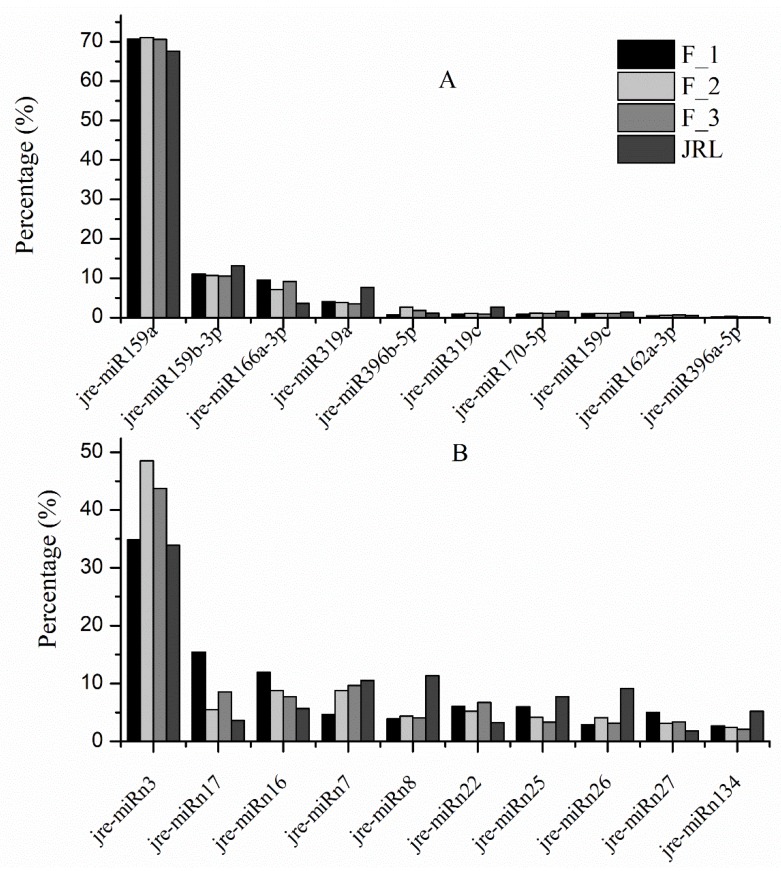
Abundance of highly expressed miRNAs in the four *J. regia* libraries. Panel (**A**) Abundance of the ten most common miRNAs as a percentage of the total known reads. Panel (**B**) Abundance of the ten most common novel miRNAs as a percentage of the total novel reads. F_1, female flower buds before flower induction; F_2, female flower buds during flower induction; F_3, female flower buds after flower induction; JRL, leaf buds during flower induction.

To identify the known miRNAs in *J. regia*, mapped sRNAs in the transcriptome were compared to other plant miRNAs in the miRBase database (release 21). The results showed that 3,348,517 total reads representing 1013 unique sRNAs matched known miRNAs. Of these unique sRNAs, 51 mature miRNAs and 60 precursors were identified (Table S1). All of the precursors were able to adopt hairpin structures resembling the fold-back structure of miRNA. The analysis of nucleotide bias of known miRNAs showed that uracil appeared with high frequency at the 5′-end (Figure S1). Among the known miRNAs, 38 miRNAs were detected in all four libraries. Two miRNAs (jre-miR170-3p and jre-miR166a-3p) were only identified in F_1. Jre-miR167c-5p was specific to F_2 and jre-miR393a-3p was specific to F_3 (Table S1). The expression levels of some miRNA families, such as MIR159 and MIR319, were much higher than those of other families (Figure 2A). The most abundant miRNA was jre-miR159a, accounting for 70.72, 71.09, 70.66 and 67.66% of the total sequences in F_1, F_2, F_3 and JRL, respectively. Jre-miR159b-3p, jre-miR166a-3p, jre-miR319a, jre-miR396b-5p, jre-miR319c and jre-miR170-5p had high expression levels, with more than 10,000 reads in each of the four libraries. Jre-miR159c, jre-miR162a-3p, and jre-miR396a-5p had moderate abundance, with more than 1000 reads. There were significant differences in expression levels among different members within the same miRNA family. For instance, in the MIR166 family, the mean expression of jre-miRNA166a-3p was 70,522 reads, whereas the mean expression of jre-miR166a-5p was only 0.25 reads. An average of 10,129 reads was obtained for jre-miR170-5p; however, only one read was identified for jre-miR170-3p. Furthermore, jre-miR166a-5p and jre-miR170-3p were only found in F_1.

### 2.3. Identification of Novel miRNAs in J. regia

Stem-loop structures, which are unique to miRNAs, were used to predict novel miRNAs in this study. A total of 371,006 novel miRNA reads were identified from the four libraries, representing 3489 unique sRNAs. A total of 75 miRNA precursors and 72 novel miRNAs were predicted from the four libraries (Table S2). The length of these potential novel miRNA precursors ranged from 65 to 295 nt. Most (47 of 72) of the novel miRNAs were located in the 5′-end of the stem-loop. The minimal folding free energy (MFE) of these precursors ranged from −14.91 to −202 kcal/mol, with an average of −54.69 kcal/mol. We also detected the complementary miRNA* sequences for each novel miRNA. The results showed that 45 of 72 novel miRNAs had complementary miRNA*s, providing more evidence that these miRNAs were real. In addition, most miRNA*s were present at lower copy numbers than those of their corresponding miRNAs. The length of the novel miRNAs ranged from 18- to 25-nt. The 21- and 24-nt miRNAs were most abundant, accounting for 37.5 and 34.72% of the total novel miRNAs, respectively. Uracil was the first nucleotide in nearly half of the novel miRNAs (Figure S1). The expression levels of most novel miRNAs were generally less than those of the known miRNAs. The exceptions were jre-miRn3, jre-miRn16 and jre-miRn17, which all had more than 10,000 reads. Figure 2B shows the ten novel miRNAs with the highest expression. The most abundant miRNA was jre-miRn3 which accounted for 34.83% of the total novel reads in F_1, 48.52% in F_2, 43.71% in F_3, and 33.88% in JRL.

A total of 24 families were identified among these known and novel miRNA precursors. These families were conserved across 69 plant species (Table S3). In *J. regia*, the biggest family was MIR159/319 with nine members, followed by MIR156/157 and MIR171/170 which both had eight members. Moreover, MIR156/157 was found in 47 plant species, making it the most common family. MIR159/319, MIR166 and MIR396 were found across 42 plant species. The MIR171_1, MIR160 and MIR172 families were identified in 37, 36, and 33 different plant species, respectively.

### 2.4. Differential Expression of miRNAs in J. regia

**Figure 3:**
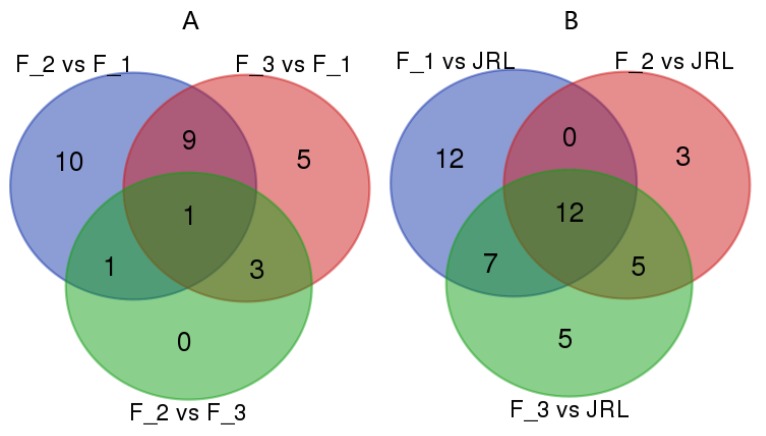
Venn diagrams of the differentially expressed miRNAs in the four libraries. (**A**) Differentially expressed miRNAs in flower buds at different stages of differentiation. (**B**) Differentially expressed miRNAs between flower buds and leaf buds. F_1, female flower buds before flower induction; F_2, female flower buds during flower induction; F_3, female flower buds after flower induction; JRL, leaf buds during flower induction.

The expression of the miRNAs was normalized so that comparisons could be made among the four libraries. The miRNAs were considered differentially expressed when |log2 (fold change)| > 1 and *q*-value < 0.005. A total of 19 known and 34 novel miRNAs were identified as differentially expressed miRNAs (Table S4). More exactly, in F_2 vs. F_1 (Figure 3A), 9 miRNAs (jre-miR395a, jre-miR396b-5p, jre-miR396a-3p, jre-miR398a-3p, jre-miRn29, jre-miRn50, jre-miRn106, jre-miRn135 and jre-miRn146) were up-regulated and 12 miRNAs (jre-miR157a-5p, jre-miR160-5P, jre-miR171b-3p, jre-miR390a-3p, jre-miRn16, jre-miRn17, jre-miRn20, jre-miRn27, jre-miRn44, jre-miRn46, jre-miRn49 and jre-miRn129) were down-regulated. In F_2 vs. F_3, 3 known miRNAs (jre-miR408-3p, jre-miR395a, and jre-miR398a-3p) were up-regulated and 2 novel miRNAs (jre-miRn147 and jre-miRn150) were down-regulated. In F_3 vs. F_1, 7 miRNAs (jre-miR396b-5p, jre-miRn29, jre-miRn41, jre-miRn50, jre-miRn146, jre-miRn147 and jre-miRn150) were up-regulated and 11 miRNAs (jre-miR160a-5p, jre-miR398a-3p, jre-miR8175, jre-miR408-3p, jre-miR390a-3p, jre-miRn17, jre-miRn20, jre-miRn25, jre-miRn122, jre-miRn129, and jre-miRn78) were down-regulated.

Among the miRNAs, jre-miR160a-5p, jre-miR171b-3p, jre-miR390a-3p, jre-miR157a-5p were down-regulated in F_2 compared to F_1 but not significantly expressed in F_3. The expressions of jre-miR396b-5p, jre-miR396b-3p, jre-miR395a, and jre-miR398a-3p were significantly up-regulated in F_2 vs. F_1. Finally, the expressions of jre-miR395a and jre-miR398a-3p were up-regulated in F_2 vs. F_3.

The expression levels of miRNAs were also compared between female flower buds (F_1, F_2, and F_3) and leaf buds (JRL) (Figure 3B). In F_1 vs. JRL, 23 miRNAs were up-regulated and eight miRNAs were down-regulated. In F_2 vs. JRL, 15 miRNAs were up-regulated and five miRNAs were down-regulated. In F_3 vs. JRL, 17 miRNAs were up-regulated and 12 miRNAs were down-regulated. The members of differentially expressed miRNAs were different in each group. Among the differentially expressed miRNAs, jre-miR166a-3p, jre-miR165a-3p, jre-miRn15, jre-miRn17, jre-miRn22, jre-miRn27, jre-miRn37, jre-miRn41, jre-miRn63 and jre-miRn69 were up-regulated whereas jre-miRn51 and jre-miR399b were down-regulated in the F_1, F_2 and F_3 libraries compared with JRL.

### 2.5. RT-qPCR Validation of J. regia miRNAs

**Figure 4:**
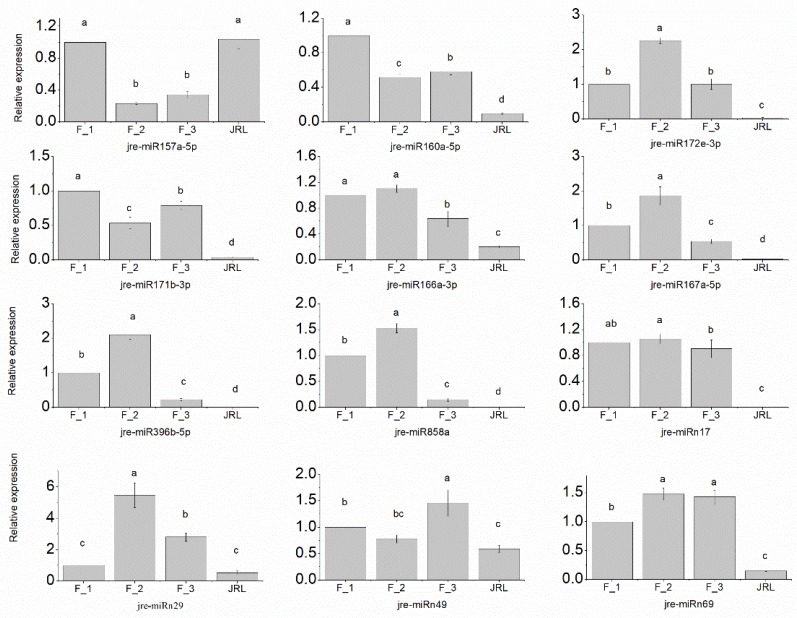
Relative expressions of 12 of the most promising miRNAs as determined by RT-qPCR. Each value is the average of three biological and technical repetitions. The error bars represent standard deviations. Values with different letters are significantly different according to Duncan’s multiple range test (*p* < 0.05).

The data from sRNA-seq was validated with RT-qPCR to determine the expression levels of ten differentially expressed miRNAs (i.e., jre-miR160a-5p, jre-miR157a-5p, jre-miR171b-3p, jre-miR396b-5p, jre-miR166a-3p, jre-miR167a-5p, jre-miRn17, jre-miRn29, jre-miRn49 and jre-miRn69) and two miRNAs (i.e., jre-miR172e-3p and jre-miR858a) with no expression difference. Jre-miR394a was used as the reference gene. As shown in Figure 4 and Table S4, the expression patterns of the selected miRNAs were consistent with those obtained from sRNA-seq data.

### 2.6. Target Prediction and Function Analysis of miRNAs in J. regia

To understand the function of the differentially expressed miRNAs, their target genes were predicted by psRobot (http://omicslab.genetics.ac.cn/psRobot/target_prediction_1.php). A total of 1339 target genes were predicted for the 19 known miRNAs and 33 novel miRNAs. No target gene was predicted for jre-miRn44 (Table S5). 

Most of the miRNAs targeted more than one gene, suggesting that these miRNAs had diverse regulatory roles. Jre-miRn3 targeted only one gene, indicating that it played a unique regulatory function. The predicted target genes were searched against the non-redundant database. A total of 616 of these target genes had an annotated function. These predicted target genes belonged to numerous transcription factors and genes with different biological functions. The target genes were involved in various functions, including floral development and flowering time. For example, floral homeotic protein *APETALA 2* (*AP2*) and ethylene-responsive transcription factor *RAP2-7* were predicted to be modulated by jre-miRn69 (Table 2 and Table S5). Squamosa promoter-binding protein 1 (*SPB1*) and squamosa promoter-binding-like protein (*SPL4/6/9/13A/16/18*) were predicted to be targeted by jre-miR157a-5p. Additionally, 16 target unigenes were involved in the hormone signaling pathway. Specifically, auxin response factor 18 (*ARF18*) was predicted to be targeted by jre-miR160a-5p, and auxin response factor 8 (*ARF8*) was predicted to be targeted by jre-miR167a-5p. Scarecrow-like protein 6 (*SCL6*) was predicted to be targeted by jre-miR171b-3p, jre-miRn46 and jre-miRn49. Jre-miRn49 was also predicted to target indole-3-acetic acid-amido synthetase *GH3.1*.

The Gene Ontology (GO) enrichment of the target genes showed that the differentially expressed miRNAs were involved in various biological processes and pathways. A total of 1612 GO terms were enriched (Table S6) and 302 significant GO terms (collected *p*-value < 0.05) were identified, including 185 “biological process” terms, 54 “cellular component” terms and 63 “molecular function” terms. Among the biological processes, most of the enriched GO terms were involved in biological regulation, including regulation of cellular process, macromolecule metabolic process, primary metabolic process, cellular metabolic process, and nitrogen compound metabolic process. For the cellular component category, the most represented GO terms were related to “cell”, “cell part”, “intracellular” and “intracellular part”. Based on the molecular function category, the represented GO terms were related to “nucleic acid binding transcription factor activity”, “transcription factor activity, sequence-specific DNA binding”, “ADP binding”, “nucleoside-triphosphatase activity” and “pyrophosphatase activity”.

**Figure 5:**
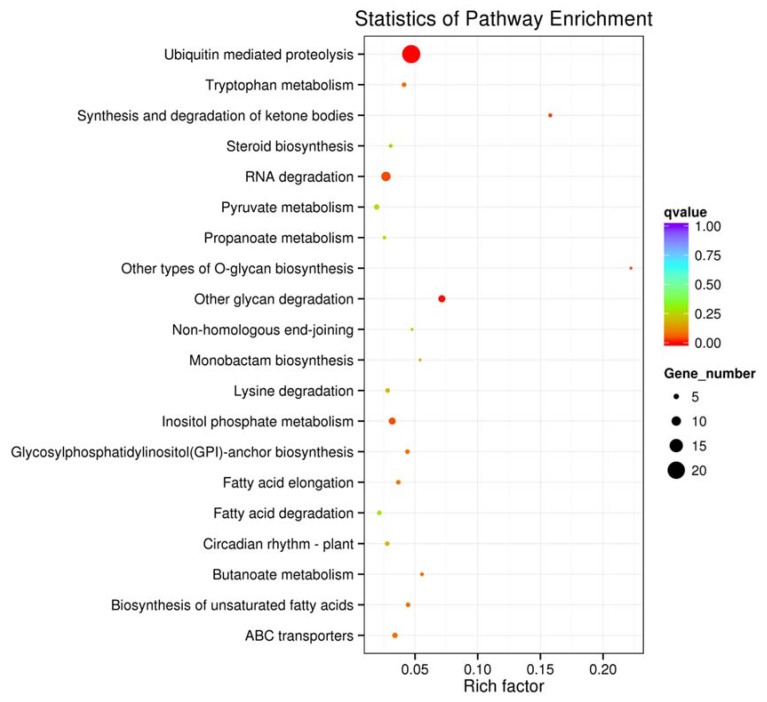
The 20 most enriched KEGG pathways of target genes of differentially expressed miRNAs. The x-axis indicates the rich factor and the y-axis indicates the pathway names.

The Kyoto Encyclopedia of Genes and Genomes (KEGG) enrichment analysis revealed 65 biochemical pathways. With the threshold of corrected *p*-value at <0.05, six pathways were significantly enriched involving 50 unigenes (Table S7). “Ubiquitin mediated proteolysis” (ko04120) was the most significantly enriched term with 21 unigenes, followed by “RNA degradation” (ko03018) with 10 unigenes, and “Other glycan degradation” (ko00511) and “Inositol phosphate metabolism” (ko00562) both with 7 unigenes (Figure 5). In addition, some pathways regulated by differentially expressed miRNAs were not significantly enriched; however they may still be important for flower induction in *J. regia*. Some examples include “plant circadian rhythm” (ko04712), “photosynthesis” (ko00195 and ko00196), “plant hormone signal transduction” (ko04075) and various carbohydrate metabolism pathways (ko00500, ko00040, ko00520, ko00030, ko00051, ko00053, ko00630, ko00650 and ko00010).

## 3. Discussion

The sRNAs ranging from 21 to 24-nt were common in this study, which was typical of mature miRNAs in plants [20,21]. The 24-nt sRNA had the highest abundance in all four libraries, accounting for an average of 50.32% of the total reads. Similar results were obtained in other species, such as *Arabidopsis* [22], *Citrus trifoliate* [23], *C. sativus* [24], *Carya cathayensis* [19], *Malus hupehensis* [25], *Pyrus bretschneideri* [26] and *Triticum turgidum* [27]. There was a difference in the number of 21-nt and 24-nt sRNAs between leaf buds (JRL) and female flower buds (F_1, F_2 and F_3) (Figure 1), suggesting that the 21-nt and 24-nt sRNAs had different regulatory functions during flower induction. The analysis of nucleotide bias showed that uracil dominated the first three positions in the known miRNAs. In addition, uracil was the first nucleotide in nearly half of the novel miRNAs (Table S1). This result was similar to previous studies about *Populus* [28], *Glycine max* [29] and *C. cathayensis* [19].

The expression of miRNA families differs across species (Table S3). In *J. regia*, jre-miR159a was the most abundant miRNA, followed by jre-miR159b-3p, jre-miR166a-3p, jre-miR319a, jre-miR396b-5p, jre-miR319c and jre-miR170-5p. All of these miRNAs had >10,000 reads, suggesting that they had important roles in flower induction. MIR159/319 was the most abundant family with nine members, followed by MIR156/157 and MIR171/170 which both had eight members. A previous report indicated that MIR166 was the largest family in *C. cathayensis* (13 members) [19] and *Cymbidium ensifolium* (eight members) [21]. In this study, MIR166 had two members. Interestingly, there were significant differences in the expression of miRNAs within the same family. For example, jre-miR166a-3p had an average of 70,522 reads, whereas jre-miR166a-5p only had one read (the read was in F_1). Jre-miR170-5p had an average of 10,129 reads, and jre-miR170-3p was only found in F_1 with one read. The large differences among miRNA family members reflect the diversity of their potential physiological roles during flower induction.

We identified 72 novel miRNAs and 45 novel miRNA*s which corresponded to 75 precursors. The lengths of these potential novel miRNA precursors ranged from 65- to 295-nt, which was close to the lengths of miRNA precursors in *Musa acuminata* [30]. The average MFE of these miRNA precursors was −54.69 kcal/mol. This value was consistent with the low MFE of miRNA precursors in *P. bretschneideri* (−51.48 kcal/mol) [26] and *Poncirus trifoliate* (−52.41kcal/mol) [18] but less than that in *C. cathayensis* (−36.9 kcal/mol) [19]. According to the criteria for annotation of novel plant miRNA, the detection of miRNA* gave strong biogenesis evidence for the novel miRNAs [31]. In this study, we detected 45 miRNA*s. The expressions of these miRNA*s were significantly lower than their corresponding miRNAs, which agreed with the previous report that miRNA* sequences were degraded and therefore usually occurred at significantly lower levels than mature miRNAs [19,32,33].

Analysis of the target genes for the differentially expressed miRNAs revealed 1339 potential unigenes. The functions of 616 of the target genes had been previously annotated, and many genes were involved in flower development and flowering time. As reported in previous studies, miR156 regulated squamosa promoter-binding (*SPB*) genes and miR172 regulated *AP2* genes [10,11]. The over-expression of miR156 prolonged the expression of juvenile vegetative traits and delayed flowering both in *Arabidopsis* [10,11,13] and in *Zea mays* [12]. In contrast, the over-expression of miR172 accelerated flowering in *Arabidopsis*. Four MIR156/157 members and five MIR172 members were identified in our study. Only jre-miR157a-5p was significantly up-regulated in F_1 vs. F_2. Jre-miRn69, which was up-regulated in F_1, F_2 and F_3 compared with JRL, was predicted to regulate *AP2* and *RAP2-7*. The result, which was validated by RT-qPCR (Figure 4), suggested that jre-miR157a-5p and jre-miRn69 both play important roles in *J. regia* female flower induction.

In *Arabidopsis*, miR167 targeted *ARF6* and *ARF8*, which regulated gynoecium and stamen development in immature flowers [34,35]. In *C. cathayensis*, cca-miR167 was up-regulated during the late stage of flower development, suggesting that the *ARF* family is important for flower development [19]. As shown in Table 2, 16 unigenes were involved in various hormone signaling pathways. The results of RT-qPCR showed that (i) jre-miR160a-5p was significantly down-regulated both in F_2 vs. F_1 and in F_3 vs. F_1 and (ii) jre-miR167a-5p was significantly up-regulated in F_1 vs. JRL. This suggests that jre-miR160a-5p and jre-miR167a-5p both play important roles during female flower induction in *J. regia*.

Transcription factor *SCL*, which belongs to the *GRAS* protein family, is targeted by miR171 and plays important roles in flowering control and apical meristem development in *Arabidopsis* [36]. In our study, *SCL6* was targeted by jre-miR171b-3p, jre-miRn46, and jre-miRn49. The result of RT-qPCR confirmed that jre-miR171b-3p and jre-miRn49 were both down-regulated in F_2 vs. F_1, suggesting that jre-miR171b-3p and jre-miRn49 may play important roles in flower induction.

## 4. Materials and Methods

### 4.1. Plant Materials

The *J. regia* (cv. Xinxin2) trees in this study were growing at the Luntai Plant Germplasm Resource Garden, Xinjiang Academy of Agricultural Science. Five trees were selected for sampling. Female flower buds were collected before (F_1), during (F_2) and after (F_3) the female flower induction. Leaf buds (JRL) were collected during female flower induction. The samples from the five trees were pooled, immediately frozen in liquid N, and then stored at −70 °C.

### 4.2. Small RNA Library Construction and Deep Sequencing

Total RNA was extracted from the female flower buds (F_1, F_2 and F_3) and leaf buds (JRL) using a Trizol kit (Invitrogen, Carlsbad, CA, USA). The plant samples for extraction each contained three flower or leaf buds. Total RNA was extracted from three samples (replications) for each flower development stage and then pooled. A 3 μg aliquot of the pooled sample of total RNA was used for sequencing. The four sequencing libraries were generated using NEBNext^®^ Multiplex Small RNA Library Prep Set for Illumina^®^ (NEB, Acton, MA, USA) following the manufacturer’s recommendations. Index codes were added to attribute sequences to each sample. Briefly, NEB 3′ SR Adaptor was directly and specifically ligated to the 3′ end of the small RNAs. After the 3′ ligation reaction, the SR RT Primer was hybridized to the excess 3′ SR Adaptor, transforming the single-stranded DNA adaptor into a double-stranded DNA molecule. Next, a 5′ end adaptor was ligated to the 5′ end of the small RNAs. The first strand cDNA was synthesized using M-MuLV Reverse Transcriptase (RNase H^−^). Next, PCR amplification was performed and the products were purified on 8% polyacrylamide gel (100 V, 80 min). The cDNA fragments with length of 140–160 bp were recovered and dissolved in 8 μL elution buffer. Finally, the quality of the cDNA libraries was assessed on the Bioanalyzer 2100 system (Agilent, Palo Alto, CA, USA) using DNA High Sensitivity Chips. The clustering of the index-coded samples was performed on a cBot Cluter Generation System using a Truseq SR Cluster Kit v3-cBot-Hs (Illumina, San Diego, CA, USA) according to the manufacturer’s instructions. The prepared libraries were sequenced on an Illumina Hiseq 2500/2000 platform. All of the processes were carried out by Novogene (Beijing, China).

### 4.3. Bioinformatics Analysis of Small RNAs

After discarding adapter contaminants and low-quality reads from the raw data, clean reads with lengths between 18 and 30-nt were chosen for analysis. The sRNAs were mapped to the *J. regia* transctriptome using the Bowtie method [37] without mismatch. Mapped sRNAs were used to search for known miRNA in the miRBase database (Release 21) (http://www.mirbase.org/). Modifications of mirdeep2 [38] and srna-tools-cli [39] software were used to obtain the potential miRNAs and to draw the secondary structures. We removed the sequences matching non-coding RNAs including rRNAs, tRNAs, snRNAs, and snoRNAs deposited in the Rfam database (http://rfam.xfam.org/). MiREvo [40] and mirdeep2 were integrated to predict novel miRNA by exploring the secondary structure, the Dicer cleavage sites, and the minimum free energy of sRNAs that were not annotated in the previous steps.

### 4.4. Expression Analysis of miRNA

The miRNA expression levels were estimated by TPM (transcript per million) using the following criteria [41]: Normalized expression = mapped read count/total reads × 1,000,000. Differential expression analysis of two samples was performed using the DEGseq (2010) R package [42]. The *p*-value was adjusted using the *q*-value. A *q*-value < 0.005 and |log2 (fold change)| > 1 were set as thresholds for significant differential expression.

### 4.5. Validation and Expression of miRNAs by RT-qPCR

The expression levels of 12 of the most promising miRNAs were determined using RT-qPCR. Total RNA samples were the same as the sRNA-seq samples. The addition of poly (A^+^) tails to sRNAs by poly (A^+^) polymerase and the synthesis of cDNA were both performed using a miRcute Plus miRNA First-strand cDNA Synthesis Kit (Tiangen, Beijing, China). The qPCRs were performed using a miRcute Plus miRNA qPCR Detection Kit (Tiangen) in a 20 μL reaction mixture consisting of 2 μL of cDNA (ten-fold dilution), 0.4 μL of 10 μM forward primer and 0.4 μL of 10 μM reverse primer and 10 μL of 2× miRcute Plus miRNA Premix (with SYBR&ROX). The reactions were carried out as follows: 95 °C for 15 min, and 40 cycles of 94 °C for 20 s and 60 °C for 34 s. Each sample was processed three times. The reference gene was chosen based on the stability analysis of eleven putative reference genes: jre-miR394a, jre-miR159a, jre-miR159c, jre-miR167d, jre-miR159b-3p, jre-miRn3, jre-miRn7, jre-miRn59, jre-miR142, U6 and 5.8s rRNA (data not shown). Jre-miR394a was the most stable gene in both the leaf buds and the female flower buds and was therefore used as the reference gene in this study. The relative expression levels of the miRNAs were quantified using the 2^−ΔΔ*C*t^ method [43]. The expression level of F_1 was set to 1. The primer sequences are listed in Table S8.

### 4.6. Target Gene Prediction and Function Annotation

The putative target genes of differentially expressed miRNAs were predicted by psRobot_tar in psRobot [44]. To determine the function of the target genes, GO enrichment analysis was performed using the GOseq R package [45]. The GO terms with corrected *p*-values < 0.05 were considered significantly enriched. Pathway analysis of the target genes was performed using the KEGG database (http://www.genome.ad.jp/kegg/) [46]. KOBAS software [47] was used to test the statistical enrichment of the target genes in the KEGG pathways at a corrected *p*-value < 0.05.

## 5. Conclusions

In summary, high-throughput sequencing was used to identify 51 known miRNAs and 72 novel miRNAs from four small RNA libraries from female flower buds and leaf buds of *J. regia*. The miRNAs belonged to 24 miRNA families and were conserved across 69 plant species. Pairwise analysis indicated that 19 of the known miRNAs and 34 of the novel miRNAs were differentially expressed. Twelve miRNAs were validated by RT-qPCR and the results were similar to those detected by sRNA-seq. A total of 1339 target genes were predicted for the differentially expressed miRNAs. The functions of 616 of the target genes have been annotated. The target genes included (i) *AP2* and ethylene-responsive transcription factor *RAP2*-7 targeted by jre-miRn69; (ii) *SPB1* and various *SPL*s targeted by jre-miR157a-5p; and (iii) various hormone responding factors targeted by jre-miR160a-5p and jre-miR167a-5p. Transcription factor *SCL6* was targeted by jre-miR171b-3p, jre-miRn46 and jre-miRn49. The KEGG pathway analysis of the target genes indicated that the differentially expressed miRNAs were mainly enriched to ubiquitin mediated proteolysis, RNA degradation and various carbohydrate metabolism pathways. These research results contribute valuable information for further research about the function of miRNAs in the flower induction of *J. regia* and other fruit trees.

## Figures and Tables

**Table 1:** Summary of small RNA sequencing and annotation in the four *J. regia* libraries. F_1, female flower buds before flower induction; F_2, female flower buds during flower induction; F_3, female flower buds after flower induction; JRL, leaf buds during flower induction.

	F_1	F_2	F_3	JRL
Raw reads	12,151,175	11,933,426	11,798,939	11,579,086
Clean reads	11,968,359	11,705,783	11,580,851	11,324,267
sRNA reads with 18–30 nt	10,765,695	10,020,557	10,094,699	8,440,666
Mapped sRNA reads	4,873,510	4,702,765	4,608,652	3,525,507
Known miRNA	964,182	828,091	952,787	603,457
Novel miRNA	129,206	87,699	95,201	58,900
rRNA, tRNA, snRNA, snoRNA, ta-siRNA	368,813	378,828	344,701	222,502
Others	3,411,309	3,408,147	3,215,963	2,640,648

**Table 2:** Flower induction-related miRNAs and target gene annotations.

MiRNA	Target Genes	Gene Description
jre-miRn69	Cluster-14922.52553, Cluster-14922.56228, Cluster-14922.99553, Cluster-14922.71416, Cluster-14922.41338, Cluster-14922.41337, Cluster-14922.50813, Cluster-14922.76833	Ethylene-responsive transcription factor RAP2-7 OS = Arabidopsis thaliana GN = RAP2-7 PE = 2 SV = 2
	Cluster-14922.29861, Cluster-14922.29860, Cluster-14922.35646, Cluster-14922.29863	Floral homeotic protein APETALA 2 OS = Arabidopsis thaliana GN = AP2 PE = 1 SV = 1
jre-miR157a-5p	Cluster-14922.56642, Cluster-14922.98238, Cluster-14922.32383, Cluster-14922.41178	Squamosa promoter-binding protein 1 OS = Antirrhinum majus GN = SBP1 PE = 2 SV = 1
	Cluster-14922.65142, Cluster-14922.65141, Cluster-14922.63070, Cluster-14922.39484	Squamosa promoter-binding-like protein 18 OS = Oryza sativa subsp. Japonica GN = SPL18 PE = 2 SV = 1
	Cluster-14922.54371, Cluster-14922.56749, Cluster-14922.54372	Squamosa promoter-binding-like protein 9 OS = Arabidopsis thaliana GN = SPL9 PE = 2 SV = 2
	Cluster-14922.69694	Squamosa promoter-binding-like protein 13A OS = Arabidopsis thaliana GN = SPL13A PE = 2 SV = 1
	Cluster-14922.29571, Cluster-14922.29570, Cluster-14922.53662, Cluster-14922.43359	Squamosa promoter-binding-like protein 6 OS = Arabidopsis thaliana GN = SPL6 PE = 2 SV = 2
	Cluster-14922.44489, Cluster-14922.44491, Cluster-14922.47505	Squamosa promoter-binding-like protein 7 OS = Oryza sativa subsp. Indica GN = SPL7 PE = 2 SV = 1
	Cluster-14922.22729, Cluster-14922.24187, Cluster-14922.24188, Cluster-14922.35309, Cluster-14922.65226	Squamosa promoter-binding-like protein 16 OS = Oryza sativa subsp. Japonica GN = SPL16 PE = 2 SV = 1
	Cluster-14922.60789, Cluster-14922.63041	Squamosa promoter-binding-like protein 4 OS = Arabidopsis thaliana GN = SPL4 PE = 1 SV = 1
jre-miR160a-5p	Cluster-14922.45926, Cluster-14922.65024, Cluster-14922.86555, Cluster-14922.65022, Cluster-14922.94990, Cluster-14922.67646 Cluster-14922.58894, Cluster-14922.92805, Cluster-14922.45431, Cluster-14922.86030, Cluster-14922.85483	Auxin response factor 18 OS = Oryza sativa subsp. Japonica GN = ARF18 PE = 2 SV = 1
jre-miR167a-5p	Cluster-14922.60064, Cluster-14922.57359, Cluster-14922.61850, Cluster-14922.62563, Cluster-14922.54061	Auxin response factor 8 OS = Arabidopsis thaliana GN = ARF8 PE = 2 SV = 2
jre-miR171b-3p, jre-miRn46, jre-miRn49	Cluster-14922.65200	Scarecrow-like protein 6 OS = Arabidopsis thaliana GN = SCL6 PE = 1 SV = 1
jre-miRn49	Cluster-14922.90636	Probable indole-3-acetic acid-amido synthetase GH3.1 OS = Arabidopsis thaliana GN = GH3.1 PE = 2 SV = 1

## Supplementary Materials

The following are available online. Figure S1: Examination of nucleotide bias within known miRNAs; Table S1: Known miRNAs in four *Juglans regia* libraries; Table S2: Novel miRNA in four *Juglans regia* libraries; Table S3: The conserved miRNA families among species; Table S4: Differentially expressed miRNAs identified by pairwise analysis; Table S5: Target gene prediction and function annotation for differentially expressed miRNAs; Table S6: GO enrichment for target genes of differentially expressed miRNAs; Table S7: KEGG pathway analysis for target genes of differentially expressed miRNAs; Table S8: Reverse transcription quantitative real-time PCR (RT-qPCR) primer sequences for candidate miRNAs.

## References

[1] Aradhya M.K., Potter D., Simon C.J. (2004). Cladistic biogeography of *Juglans* (*Juglandaceae*) based on chloroplast DNA intergenic spacer sequences. Darwins Harvest New Approaches to the Origins.

[2] Xi R. (1990). Discussion on the origin of walnut in China. Acta Hortic..

[3] Valiente J.I., Albrigo L.G. (2004). Flower bud induction of sweet orange trees [*Citrus Sinensis* (L.) Osbeck]: Effect of low temperatures, crop load, and bud age. J. Am. Soc. Hortic. Sci..

[4] Ben-Tal Y. (1986). Flowering: Its control by vegetative growth inhibition. V International Symposium on Growth Regulators in Fruit Production.

[5] Xia X., Xi R. (1989). The periods of physiological and morphological differentiation of pistillate flower buds in walnut (*Juglans regia* L.). J. Agric. Univ. Hebei.

[6] Khan M.R.G., Ai X.Y., Zhang J.Z. (2014). Genetic regulation of flowering time in annual and perennial plants. Wiley Interdiscip. Rev. RNA.

[7] Ambros V. (1993). The *C. elegans* heterochronic gene *lin*-4 encodes small RNAs with antisense complementarity to *lin*-14. Cell.

[8] Reinhart B.J., Slack F.J., Basson M., Pasquinelli A.E., Bettinger J.C., Rougvie A.E., Horvitz H.R., Ruvkun G. (2000). The 21-nucleotide *let*-7 RNA regulates developmental timing in *Caenorhabditis elegans*. Nature.

[9] Eleonora S., Stephen J. (2014). The role of microRNAs in the control of flowering time. J. Exp. Bot..

[10] Chen X. (2004). A microRNA as a translational repressor of *APETALA2* in *Arabidopsis* flower development. Science.

[11] Aukerman M.J., Sakai H. (2003). Regulation of flowering time and floral organ identity by a microRNA and its *APETALA2*-like target genes. Plant Cell.

[12] Chuck G., Meeley R., Irish E., Sakai H., Hake S. (2007). The maize *tasselseed4* microRNA controls sex determination and meristem cell fate by targeting *Tasselseed6*/*indeterminate spikelet1*. Nat. Genet..

[13] Wu G., Park M.Y., Conway S.R., Wang J.W., Weigel D., Poethig R.S. (2009). The sequential action of miR156 and miR172 regulates developmental timing in *Arabidopsis*. Cell.

[14] Terzi L.C., Simpson G.G. (2008). Regulation of flowering time by RNA processing. Curr. Top. Microbiol. Immunol..

[15] Jones-Rhoades M.W., Bartel D.P., Bartel B. (2006). MicroRNAs and their regulatory roles in plants. Annu. Rev. Plant Biol..

[16] Fahlgren N., Montgomery T.A., Howell M.D., Allen E., Dvorak S.K., Alexander A.L., Carrington J.C. (2006). Regulation of auxin response factor3 by TAS3 ta-siRNA affects developmental timing and patterning in *Arabidopsis*. Curr. Biol..

[17] Kim W., Ahn H.J., Chiou T.J., Ahn J.H. (2011). The role of the miR399-PHO2 module in the regulation of flowering time in response to different ambient temperatures in *Arabidopsis thaliana*. Mol. Cells.

[18] Zhang J.Z., Ai X.Y., Guo W.W., Peng S.A., Deng X.X., Hu C.G. (2012). Identification of miRNAs and their target genes using deep sequencing and degradome analysis in *Trifoliate orange* [*Poncirus trifoliate* (L.) Raf]. Mol. Biotechnol..

[19] Wang Z.J., Huang J.Q., Huang Y.J., Li Z., Zheng B.S. (2012). Discovery and profiling of novel and conserved microRNAs during flower development in *Carya cathayensis* via deep sequencing. Planta.

[20] Zhao M., Chen L., Wang T., Tian Q., Zhang W.H. (2011). Identification of drought-responsive microRNAs in *Medicago truncatula* by genome-wide high-throughput sequencing. BMC Genom..

[21] Li X., Jin F., Jin L., Jackson A., Ma X., Shu X., Wu D., Jin G. (2015). Characterization and comparative profiling of the small RNA transcriptomes in two phases of flowering in *Cymbidium ensifolium*. BMC Genom..

[22] Rajagopalan R., Vaucheret H., Trejo J., Bartel D.P. (2006). A diverse and evolutionarily fluid set of microRNAs in *Arabidopsis thaliana*. Genes Dev..

[23] Song C., Chen W., Zhang C., Korir N.K., Yu H., Ma Z., Fang J. (2010). Deep sequencing discovery of novel and conserved microRNAs in *Trifoliate orange* (*Citrus trifoliata*). BMC Genom..

[24] Kou S.J., Wu X.M., Liu Z., Liu Y.L., Xu Q., Guo W.W. (2012). Selection and validation of suitable reference genes for miRNA expression normalization by quantitative RT-PCR in citrus somatic embryogenic and adult tissues. Plant Cell Rep..

[25] Xing L., Zhang D., Li Y., Zhao C., Zhang S., Shen Y., An N., Han M. (2014). Genome-wide identification of vegetative phase transition-associated microRNAs and target predictions using degradome sequencing in *Malus hupehensis*. BMC Genom..

[26] Wu J., Wang D., Liu Y., Wang L., Qiao X., Zhang S. (2014). Identification of miRNAs involved in pear fruit development and quality. BMC Genom..

[27] Paola D.D., Zuluaga D.L., Sonnante G. (2016). The miRNAome of durum wheat: Isolation and characterisation of conserved and novel microRNAs and their target genes. BMC Genom..

[28] Barakat A., Wall P.K., Diloreto S., Depamphilis C.W., Carlson J.E. (2007). Conservation and divergence of microRNAs in *Populus*. BMC Genom..

[29] Kulcheski F.R., Oliveira L.F.D., Molina L.G., Almerão M.P., Rodrigues F.A., Marcolino J., Barbosa J.F., Stolf-Moreira R., Nepomuceno A.L., Marcelino-Guimarães F.C. (2011). Identification of novel soybean microRNAs involved in abiotic and biotic stresses. BMC Genom..

[30] Bi F., Meng X., Ma C., Yi G. (2015). Identification of miRNAs involved in fruit ripening in cavendish bananas by deep sequencing. BMC Genom..

[31] Meyers B.C., Axtell M.J., Bartel B., Bartel D.P., Baulcombe D., Bowman J.L., Cao X., Carrington J.C., Chen X., Green P.J. (2008). Criteria for annotation of plant microRNAs. Plant Cell.

[32] Creighton C.J., Benham A.L., Zhu H., Khan M.F., Reid J.G., Nagaraja A.K., Fountain M.D., Dziadek O., Han D., Ma L. (2010). Discovery of novel microRNAs in female reproductive tract using next generation sequencing. PLoS ONE.

[33] Zhao Y.T., Wang M., Fu S.X., Yang W.C., Qi C.K., Wang X.J. (2012). Small RNA profiling in two *Brassica napus* cultivars identifies microRNAs with oil production-and development-correlated expression and new small RNA classes. Plant Physiol..

[34] Ru P., Xu L., Ma H., Huang H. (2006). Plant fertility defects induced by the enhanced expression of microRNA167. Cell Res..

[35] Wu M.F., Tian Q., Reed J.W. (2006). *Arabidopsis* microRNA167 controls patterns of ARF6 and ARF8 expression, and regulates both female and male reproduction. Development.

[36] Lee M.H., Kim B., Song S.K., Heo J.O., Yu N.I., Lee S.A., Kim M., Kim D.G., Sohn S.O., Lim C.E. (2008). Large-scale analysis of the gras gene family in *Arabidopsis thaliana*. Plant Mol. Biol..

[37] Langmead B., Trapnell C., Pop M., Salzberg S.L. (2009). Ultrafast and memory-efficient alignment of short DNA sequences to the human genome. Genome Biol..

[38] Friedländer M.R., Mackowiak S.D., Li N., Chen W., Rajewsky N. (2012). miRDeep2 accurately identifies known and hundreds of novel microRNA genes in seven animal clades. Nucleic Acids Res..

[39] Moxon S., Schwach F., Dalmay T., Maclean D., Studholme D.J., Moulton V. (2008). A toolkit for analysing large-scale plant small RNA datasets. Bioinformatics.

[40] Ming W., Yang S., Shi S., Tian T. (2012). miREvo: An integrative microRNA evolutionary analysis platform for next-generation sequencing experiments. BMC Bioinform..

[41] Zhou L., Chen J., Li Z., Li X., Hu X., Huang Y., Zhao X., Liang C., Wang Y., Sun L. (2010). Integrated profiling of microRNAs and mRNAs: MicroRNAs located on Xq27.3 associate with clear cell renal cell carcinoma. PLoS ONE.

[42] Wang L., Feng Z., Wang X., Wang X., Zhang X. (2010). Degseq: An R package for identifying differentially expressed genes from RNA-seq data. Bioinformatics.

[43] Livak K.J., Schmittgen T.D. (2001). Analysis of relative gene expression data using real-time quantitative PCR and the 2^−ΔΔ*C*T^ method. Methods.

[44] Wu H.J., Ma Y.K., Chen T., Wang M., Wang X.J. (2012). Psrobot: A web-based plant small RNA meta-analysis toolbox. Nucleic Acids Res..

[45] Young M.D., Wakefield M.J., Smyth G.K., Oshlack A. (2010). Gene ontology analysis for RNA-seq: Accounting for selection bias. Genome Biol..

[46] Kanehisa M., Araki M., Goto S., Hattori M., Hirakawa M., Itoh M., Katayama T., Kawashima S., Okuda S., Tokimatsu T. (2008). Kegg for linking genomes to life and the environment. Nucleic Acids Res..

[47] Mao X., Cai T., Olyarchuk J.G., Wei L. (2005). Automated genome annotation and pathway identification using the KEGG Orthology (KO) as a controlled vocabulary. Bioinformatics.

