# Supplementary material for: Identification and Expression of miRNAs Related to Female Flower Induction in Walnut (Juglans regia L.)
Source: Molecules. 2018 May 17;23(5):1202. doi: 10.3390/molecules23051202 (PMC6099546; doi:10.3390/molecules23051202)

Figure S1. Examination of nucleotide bias within known miRNAs. (a) and novel miRNAs (b).

Bar height is proportional to the frequency of the corresponding base at the given position from 1 to 22.


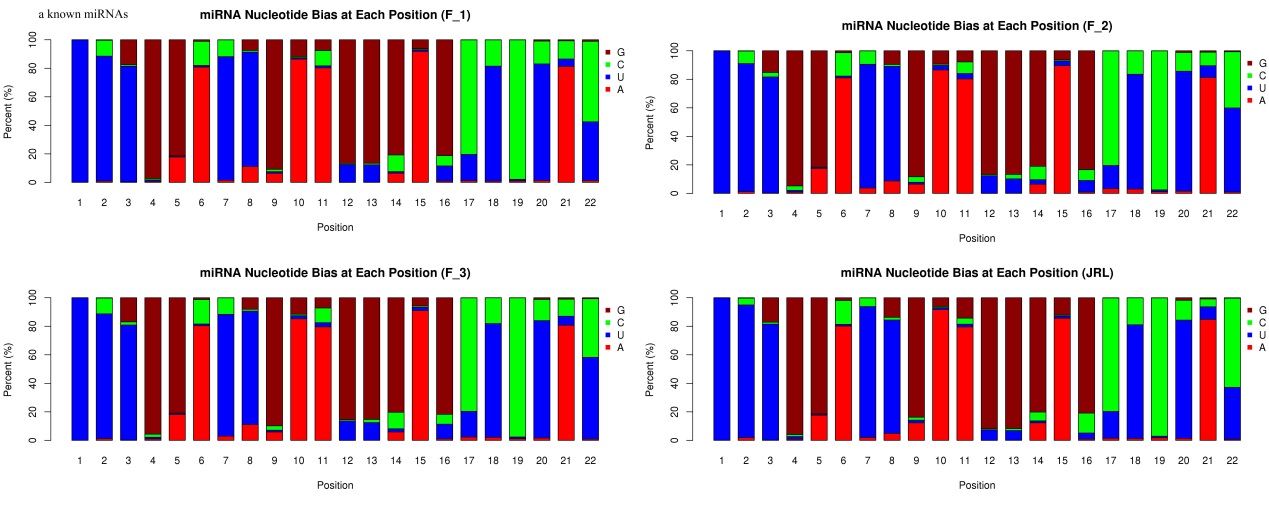


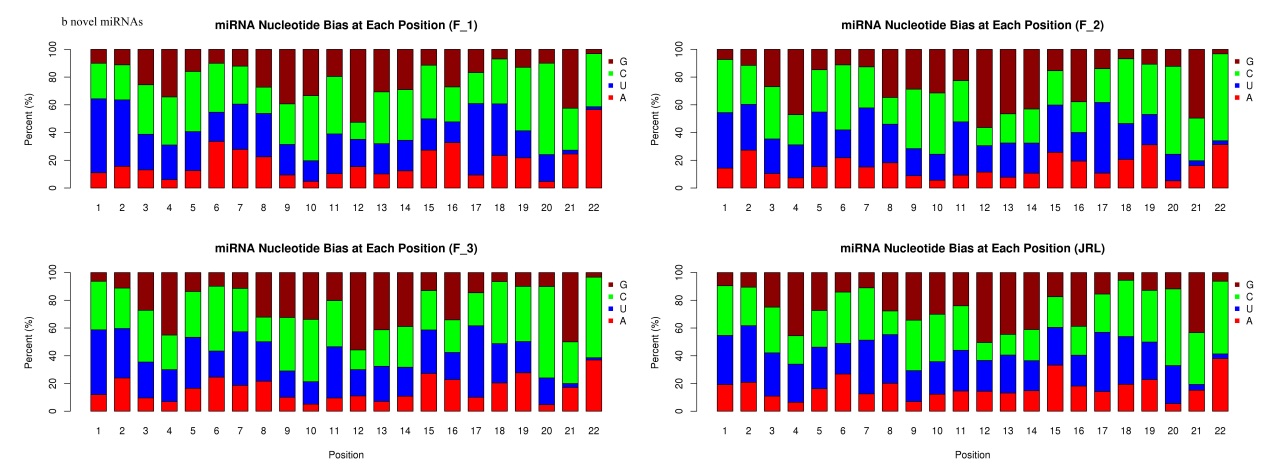

Supplement: Supplementary file 1 [file molecules-23-01202-s001.zip › Supplemental files/Figure S1.docx]
